# Inorganic Phosphate Prevents Erk1/2 and Stat3 Activation and Improves Sensitivity to Doxorubicin of MDA-MB-231 Breast Cancer Cells

**DOI:** 10.3390/molecules200915910

**Published:** 2015-09-01

**Authors:** Luigi Sapio, Luca Sorvillo, Michela Illiano, Emilio Chiosi, Annamaria Spina, Silvio Naviglio

**Affiliations:** Department of Biochemistry, Biophysics and General Pathology, Medical School, Second University of Naples, via L. De Crecchio 7, 80138 Naples, Italy; E-Mails: sapioluigi@hotmail.it (L.S.); lucasorvillo@hotmail.com (L.S.); michela_illiano@libero.it (M.I.); emilio.chiosi@unina2.it (E.C.); annamaria.spina@unina2.it (A.S.)

**Keywords:** inorganic phosphate, doxorubicin, combination antitumor therapy, Erk1/2, Stat3

## Abstract

Due to its expression profile, triple-negative breast cancer (TNBC) is refractory to the most effective targeted therapies available for breast cancer treatment. Thus, cytotoxic chemotherapy represents the mainstay of treatment for early and metastatic TNBC. Therefore, it would be greatly beneficial to develop therapeutic approaches that cause TNBC cells to increase their sensitivity to cytotoxic drugs. Inorganic phosphate (Pi) is emerging as an important signaling molecule in many cell types. Interestingly, it has been shown that Pi greatly enhances the sensitivity of human osteosarcoma cell line (U2OS) to doxorubicin. We investigated the effects of Pi on the sensitivity of TNBC cells to doxorubicin and the underlying molecular mechanisms, carrying out flow cytometry-based assays of cell-cycle progression and cell death, MTT assays, direct cell number counting and immunoblotting experiments. We report that Pi inhibits the proliferation of triple-negative MDA-MB-231 breast cancer cells mainly by slowing down cell cycle progression. Interestingly, we found that Pi strongly increases doxorubicin-induced cytotoxicity in MDA-MB-231 cells by apoptosis induction, as revealed by a marked increase of sub-G1 population, Bcl-2 downregulation, caspase-3 activation and PARP cleavage. Remarkably, Pi/doxorubicin combination-induced cytotoxicity was dynamically accompanied by profound changes in Erk1/2 and Stat3 protein and phosphorylation levels. Altogether, our data enforce the evidence of Pi acting as a signaling molecule in MDA-MB-231 cells, capable of inhibiting Erk and Stat3 pathways and inducing sensitization to doxorubicin of TNBC cells, and suggest that targeting Pi levels at local sites might represent the rationale for developing effective and inexpensive strategies for improving triple-negative breast cancer therapy.

## 1. Introduction

Triple-negative breast cancers (TNBCs) have several characteristic aggressive clinico-pathological features, including young age at onset and large tumor size, and they constitute approximately 20% of all diagnosed breast cancer cases [1]. They are heterogeneous cancers, but share an absence of estrogen receptor (ER), progesterone receptor (PR) and human epidermal growth factor receptor 2 (HER2) in their tumor cells. Its histological characteristics include high grade, high proliferative activity, focal areas of necrosis, absence of infiltrative margin, absence of gland formation, presence of central scar/fibrotic foci and prominent lymphoplasmacytic inflammatory infiltrate [2].

Because of its expression profile, there are currently no effective targeted therapies for TNBC treatment, and systemic treatment options are mainly limited to cytotoxic chemotherapy [3,4].

However, despite chemotherapy, TNBCs relapse very frequently and aggressively, with distant metastases occurring mainly in lung, bone, liver and brain, so that TNBCs have a poor prognosis in terms of disease-free and overall survival [5]. Thus, new pharmacological approaches for TNBC patients are warranted.

Combination chemotherapy has received more attention in order to find compounds that could increase the therapeutic index of clinical anticancer drugs while limiting their potential toxicity [6,7]. Doxorubicin is a potent chemotherapeutic agent, and its use is part of several standard regimens for different cancers, including TNBCs [3,8]. Although doxorubicin exerts robust antitumor activity, its effectiveness is often limited by drug-resistance and relevant dose-dependent side effects, especially doxorubicin-induced cardiotoxicity [9,10,11]. Therefore, novel combination chemotherapeutic strategies, in which one novel compound is added to increase the therapeutic index of doxorubicin, would definitely benefit cancer patients.

In this regard, naturally-occurring molecules with no or the least toxicity to normal tissues and able to reduce the dosage of doxorubicin required to obtain antitumor effects are suggested as very attractive candidates [12,13,14,15]. Inorganic phosphate (Pi) is an essential nutrient to the living organisms. It is required as a component of energy metabolism, kinase/phosphatase signaling and in the formation and function of lipids, carbohydrates and nucleic acids, and at systemic level, it plays a key role in normal skeletal and dentin mineralization [16]. Pi represents an abundant dietary element, and its intestinal absorption is efficient and minimally regulated. The maintenance of proper Pi homeostasis is a critical event; the kidney is a major regulator of Pi homeostasis and can increase or decrease its Pi reabsorptive capacity to accommodate the Pi need [17]. Interestingly, Pi is emerging as an important signaling molecule capable of modulating multiple cellular functions by altering signal transduction pathways, gene expression and protein abundance in many cell types, including TNBCs [18,19,20,21]. Importantly, it has been described that in osteosarcoma cells, Pi is capable of inducing sensitization to doxorubicin via p53-dependent apoptosis and through a mechanism involving Erk1/2 downregulation [22].

The purpose of this study was to investigate the possible effects of Pi on the chemosensitivity to doxorubicin of TNBC cells and the underlying mechanisms.

## 2. Results and Discussion

### 2.1. Pi Inhibits the Proliferation of Human MDA-MB-231 Triple-Negative Breast Cancer Cells

The human triple-negative MDA-MB-231 and estrogen and progesterone receptor-positive MCF-7 breast cancer cell lines are well-established and widely used model systems of breast cancer cells.

Recently, we started to evaluate the consequences of elevated Pi on the behavior of MDA-MB-231 breast cancer cells [19]. Here, first, we looked at the impact of inorganic phosphate on the proliferation of both MDA-MB-231 and MCF-7 cells.

For this purpose, we performed dose-response and time-course experiments. Throughout our experiments, we have used a spectrum of final concentration of Pi in agreement with most of the published studies on Pi-triggered effects [23,24,25].

**Figure 1:**
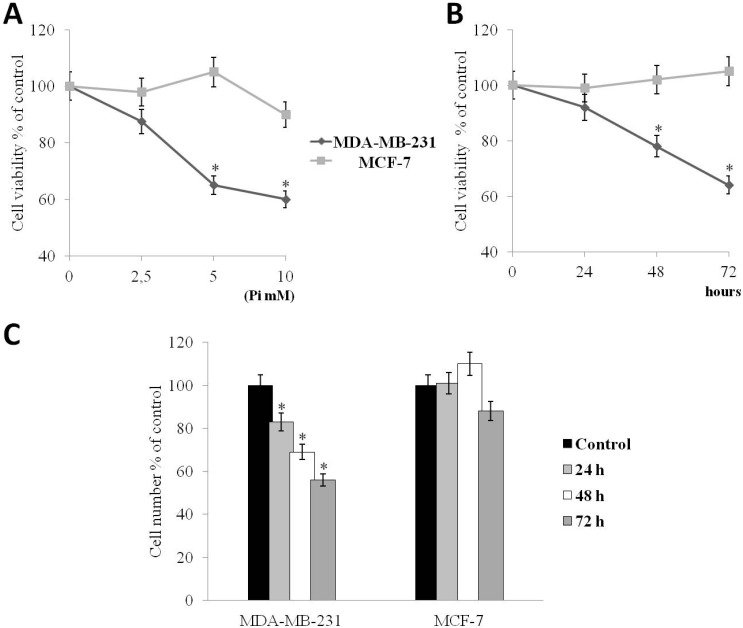
Effects of inorganic phosphate (Pi) on the proliferation of MDA-MB-231 and MCF-7 breast cancer cells. (**A**) Dose-response. MDA-MB-231 and MCF-7 cells were cultured in medium supplemented with 2.5, 5 and 10 mM Pi or not (control) for 72 h; (**B**) Time-course. MDA-MB-231 and MCF-7 cells were cultured in medium supplemented with 5 mM Pi or not (control) for 24, 48, 72 h. Then, cell viability was measured by the MTT assay; (**C**) Time-course. MDA-MB-231 and MCF-7 cells were plated at 5 × 10^5^/10-cm plate, cultured in medium supplemented with 5 mM Pi or not (control) for 24, 48, 72 h and the cell number recorded. Data represent the average of three independent experiments. The means and SD are shown. * *p* < 0.05 *vs.* control untreated cells.

MDA-MB-231 and MCF-7 cells were incubated with increasing (2.5, 5, 10 mM) concentrations of Pi for 72 h, and then, cell proliferation was determined by the conventional MTT assay (Figure 1A) and by direct cell number counting (data not shown).

Figure 1A shows that Pi causes a statistically-significant reduction of cell viability of MDA-MB-231 cells (*p* < 0.05) in a dose-dependent manner of 12%, 35%, 40% at 2.5, 5, 10 mM concentrations, respectively. Next, we performed time-course experiments. MDA-MB-231 and MCF-7 cells were exposed to 5 mM Pi (sub-maximal dose) for 24, 48 and 72 h, after which cell proliferation was determined by the conventional MTT assay and by direct cell number counting (Figure 1B,C). Figure 1B shows that Pi causes a statistically-significant reduction of the cell viability of MDA-MB-231 cells (*p* < 0.05) of 12%, of 24%, of 36% at 24, 48, 72 h, respectively. Parallel direct cell counting and growth curves provided similar results (Figure 1C).

Figure 1, in all panels, shows that the growth inhibitory effect in response to Pi was not clearly evident in MCF-7 cells.

### 2.2. Pi Causes a Slowing Down of the Cell Division Cycle in MDA-MB-231 Cells

**Figure 2:**
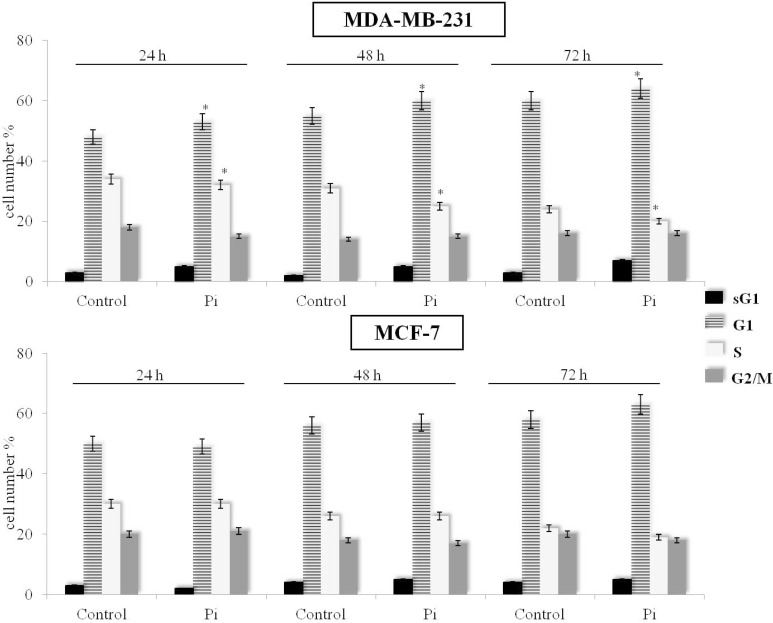
Effects of Pi on the distribution of MDA-MB-231 and MCF-7 cells in the cell cycle and sub-G1 phases. Cells were cultured in medium supplemented with 5 mM Pi or not (control) for 24, 48, 72 h. Then, FACS analysis of propidium iodide-stained cells was performed. Quantitative data indicating the percentage of hypoploid sub-G1, G1, S and G2/M MDA-MB-231 (**top**) and MCF-7 (**bottom**) cells from three independent experiments are shown. The means and SD are shown. * *p* < 0.05 *vs.* control untreated cells.

In order to evaluate the effect of Pi on MDA-MB-231 cells, we determined its possible effect on the cell cycle distribution. Cell cycle was evaluated by FACS analysis of propidium iodide-stained cells. As previously demonstrated [19], we confirm that Pi-treated MDA-MB-231 cells show a higher percentage in the G1 phase and a lower percentage in the S phase compared to control cells from 24 up to 72 h (*p* < 0.05). Moreover, only at 72 h, a small appearance of the sub-G1 population in response to Pi treatment was observed (Figure 2, top).

In Figure 2, bottom, it is shown that, in contrast to MDA-MB-231 cells, no obvious changes on cell cycle distribution in response to Pi can be seen in MCF-7 breast cancer cells up to 72 h.

Overall, the above data suggest that the anti-proliferative effect caused by inorganic phosphate in MDA-MB-231 and not in MCF-7 breast cancer cells is mainly due to a slowing down of the cell division cycle (and not due to apoptosis induction) and that Pi can have discrete effects on the cell cycle depending on the cell type/cellular background.

### 2.3. Pi Enhances Doxorubicin-Induced Cytotoxicity in MDA-MB-231 Cells

Doxorubicin is a DNA-damaging agent that generates DNA double-strand breaks (DNA DSBs) by inhibiting topoisomerase II [26]. Doxorubicin is largely used as a relevant antitumor drug widely included in standard regimens for treating breast cancer, as well as other tumors [3,8,9].

**Figure 3:**
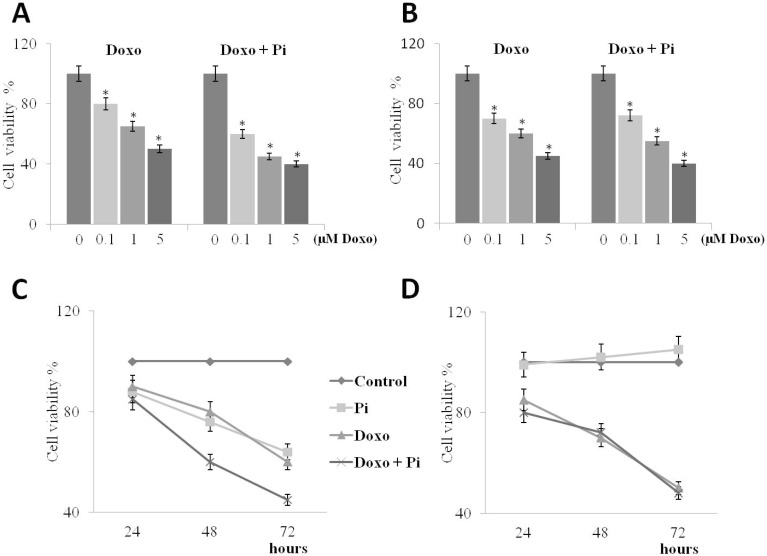
Effects of Pi on doxorubicin-induced cytotoxicity. Dose-response (**A**,**B**) and time-course (**C**,**D**) experiments. MDA-MB-231 (**A**) and MCF-7 (**B**) cells were treated or not with increasing concentrations of doxorubicin alone or in combination with 5 mM Pi for 48 h. Then, the cell viability was measured by the MTT assay. Data represent the average of three independent experiments. The means and SD are shown. * *p* < 0.05 *vs.* control untreated cells. MDA-MB-231 (**C**) and MCF-7 (**D**) cells were treated or not with 0.1 μM doxorubicin alone or in combination with 5 mM Pi for 24, 48 and 72 h. Then, the cell viability was measured by the MTT assay. Data represent the average of three independent experiments. The means and SD are shown.

We then investigated whether Pi could enhance doxorubicin-induced cytotoxicity in breast cancer cells. For this purpose, we treated MDA-MB-231 and MCF-7 cells with varying concentrations of doxorubicin, in the presence or absence of 5 mM Pi. Specific treatment conditions were examined encompassing exposure to no (0 μM, control), very low (0.1 μM), low (1 μM) or high (5 μM) doxorubicin, in the presence or absence of 5 mM Pi for 48 h (Figure 3A,B).

After treatments, a conventional tetrazolium-based (MTT) assay was performed. As expected, proliferation of both MDA-MB-231 and MCF-7 cells was slightly inhibited by doxorubicin in a dose-dependent manner [13,27,28,29].

Interestingly, we found that in MDA-MB-231 cells, the presence of Pi strongly enhanced the anti-proliferative effects of doxorubicin in all combinations, whereas no further increase of the doxorubicin-induced cytotoxicity in response to Pi could be seen in MCF-7 cells. To note, at very low dose 0.1 μM doxorubicin, the growth inhibition in triple-negative MDA-MB-231 cells increases from 20% to 40% in the presence of Pi (Figure 3A).

Moreover, we also looked at Pi/doxorubicin combination-induced cytotoxicity during a time course.

Cells were exposed to no (0 μM, control) or very low (0.1 μM) doxorubicin in the presence or absence of 5 mM Pi for 24, 48 and 72 h (Figure 3C,D).

Thereafter, the MTT assay was performed. As expected, cell viability was decreased by doxorubicin in a time-dependent manner in both cell lines. Remarkably, the growth inhibitory effects induced by the Pi/doxorubicin combination are significantly higher than those caused by doxorubicin alone in MDA-MB-231 and not in MCF-7 cells.

Overall, the above data suggest that Pi enhances doxorubicin-induced cytotoxicity in triple-negative MDA-MB-231 and does not in “not triple-negative” MCF-7 breast cancer cells, very likely according to the different cell type/cellular background.

**Figure 4:**
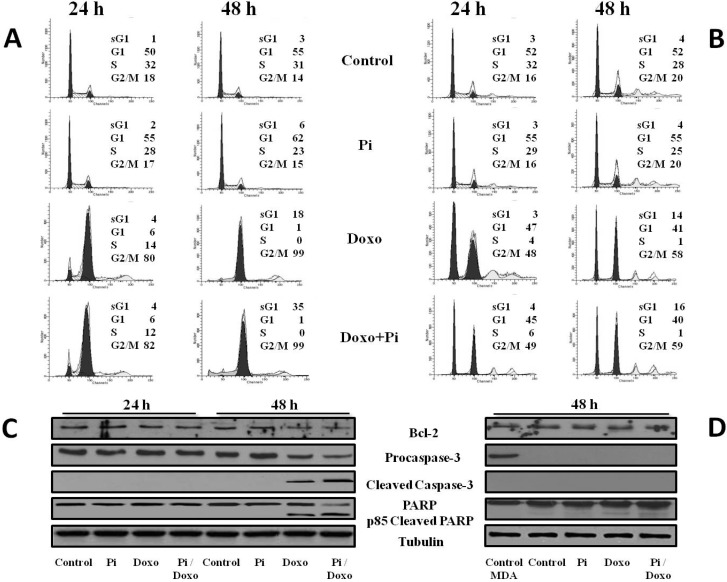
Effects of Pi, doxorubicin and Pi/doxorubicin combination on the apoptosis of MDA-MB-231 and MCF-7 cells. Treatments with Pi 5 mM, doxorubicin 0.1 μM, Pi 5 mM/doxorubicin 0.1 μM were carried out for 24 and 48 h. Representative FACS histograms of propidium iodide-stained MDA-MB-231 (**A**) and MCF-7 (**B**) cells (20,000 events/sample) are shown. The percentage of hypoploid sub-G1 and of each cell-cycle phases is indicated; (**C**) MDA-MB-231 and (**D**) MCF-7; the effects on procaspase-3, cleaved caspase-3, PARP and Bcl-2 protein levels are shown. In (**D**) is included also a lane referring to MDA-MB-231 cells used as the internal control. Thirty micrograms of cell extracts from cells treated for the indicated times were subjected to SDS-PAGE and blotted with antibodies against the indicated proteins (tubulin was used as a standard for the equal loading of protein in the lanes). The image is representative of three immunoblotting analyses from three different cellular preparations with similar results.

Since a possible explanation of the differential sensitivity of MDA-MB-231 and MCF-7 cells to Pi could be the status of caspase-3 (MDA-MB-231 and MCF-7 cells are caspase-3 positive and deficient, respectively; see also Figure 4C,D, we tested the effects of Pi also on other two different human breast cancer cell lines T-47D and ZR-75-1(these cell lines present different receptors status, but both are caspase-3-positive). Importantly, we found that in both of these two caspase-3-positive breast cancer cell lines, Pi did not enhance doxorubicin-induced cytotoxicity (exactly as in MCF-7 cells that, on the contrary, are caspase-3-deficient), strongly suggesting that the differential sensitivity of MDA-MB-231 and MCF-7 cells to Pi is not due to the caspase-3 status (data not shown).

### 2.4. Pi Potentiates the Doxorubicin-Induced Cytotoxicity in MDA-MB-231 Cells by Inducing Apoptosis

To better understand the enhancement by Pi of doxorubicin-induced cytotoxicity, MDA-MB-231 cells were exposed to no (0 μM, control) and very low (0.1 μM) doxorubicin in the presence or absence of 5 mM Pi for 24 and 48 h. Then, cells were fixed and used for flow cytometry-based assays of cell-cycle progression and cell death.

In agreement with previous results [22,30], Figure 4A shows that doxorubicin-treated MDA-MB-231 cells strongly accumulated in the G2 phase with a concomitant decrease of the G1 and S phases of the cell cycle; G2 accumulation was already evident at 24 h (almost 80% of cells in G2) and increased at 48 h (more than 90% of cells in G2). Moreover, a sub-G1 population (18%) in response to doxorubicin was observed at 48 h. Interestingly, the Doxo/Pi combination induced a further increase of the sub-G1 population at 48 h up to 35%.

To increase the specificity and reliability of these data, we performed similar experiments in MCF-7 cells, as well. According to the lack of additive effects by Pi on the doxorubicin-induced cytotoxicity in MCF-7 cells, Figure 4B shows no increase of the sub-G1 population in response to the Doxo/Pi combination compared to treatment with doxorubicin alone. Moreover, in agreement with the different sensitivity to doxorubicin and cellular heterogeneity of the two breast cancer cell types, in Figure 4B, it is also shown that the cell cycle profiles in response to doxorubicin are completely different in MCF-7 compared to MDA-MB-231 cells [27,28,29].

Accumulation of MDA-MB-231 cells with a hypodiploid DNA content (Figure 4A) is consistent with cell death by apoptosis. This was further confirmed by examining the activation of the terminal caspase-3, executioner of apoptosis and cleavage of poly(ADP-ribose)polymerase, PARP, a known target for apoptosis-associated caspase cleavage [31,32].

Figure 4C shows a decrease of the uncleaved isoform of caspase-3 and the appearance of the cleaved isoform of caspase-3 in 48-h Doxo-treated and, even more, Doxo/Pi-treated MDA-MB-231 cells, suggesting the increase of its activity that is correlated to its fragmentation. Moreover, we have evaluated the effects of the different treatments on the fragmentation of PARP that is a substrate for caspase-3. The pattern of the PARP processing paralleled that of caspase-3 cleavage (Figure 4C). In addition, Bcl2 anti-apoptotic protein was consistently downregulated in Doxo/Pi-treated MDA-MB-231 cells [33].

According to the lack of induction of the sub-G1 population in response to the Doxo/Pi combination compared to the treatment with doxorubicin alone, Figure 4D shows that in MCF-7 cells, no obvious variations on PARP and Bcl2 protein levels occur in response to Doxo/Pi co-treatment. Moreover, in Figure 4D, it is also shown that, consistent with numerous reports indicating that MCF-7 cells are caspase-3-deficient, no procaspase-3 could be detected in MCF-7 cell extracts.

Overall, the above data suggest that Pi potentiates the Doxo-induced antiproliferative effects by inducing apoptosis of G2-arrested MDA-MB-231 cells.

### 2.5. Pi Relevantly Affects Erk1/2 and Stat3 Protein and Phosphorylation Levels in Response to Doxorubicin in MDA-MB-231 Cells

The extracellular signal-regulated kinase (Erk)- and signal transducer and activator of transcription 3 (Stat3)-dependent signaling pathways are relevant to breast cancer, and several studies demonstrate that they are frequently activated [34,35].

In addition, it is largely known that in several types of cancers, including breast cancer, Erk1/2 are upregulated in response to DNA-damaging chemotherapeutic agents, such as cisplatin or doxorubicin [28,29,36,37], and also, Stat3 protein has been described to be further activated upon doxorubicin treatment in breast cancer cells [27].

**Figure 5:**
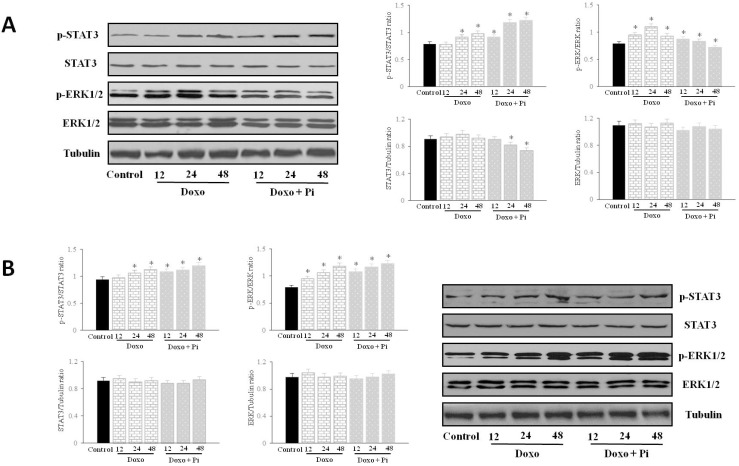
Effects of Pi, doxorubicin and Pi/doxorubicin combination on Erk1/2 and Stat3 activation. MDA-MB-231 (**A**) and MCF-7 (**B**) cells were treated or not with 0.1 μM doxorubicin alone or in combination with 5 mM Pi during a time course for up 48 h. The activation (phosphorylation) and levels of Erk1/2 and Stat3 proteins were assessed by Western blotting from 30 μg of cell extracts using antibodies against the indicated proteins. The image is representative of three different experiments with similar results. Graphs showing the densitometric intensity of pErk/Erk, pStat3/Stat3, Erk/tubulin, Stat3/tubulin bands ratio are shown. The intensities of signals were expressed as arbitrary units. * *p* < 0.05 *vs.* control untreated cells.

In order to investigate the possible role of Erk1/2 and/or Stat3 in the potentiation by Pi of Doxo-induced cytotoxicity in MDA-MB-231 cells, Western blotting was applied to examine the expression and phosphorylation (activation) of Erk1/2 and Stat3 proteins from extracts of cells treated with Pi, doxorubicin and Pi/doxorubicin combination during a time course for up to 48 h (Figure 5). To note, previously, we have provided evidence that Pi 5 mM treatment in MDA-MB-231 cells resulted in a strong inhibition of Erk 1/2 phosphorylation starting at 24 h and maintained for up to 72 h without obvious variations in the total amount of Erk1/2 protein levels and also in a dramatic decrease of the total amount of Stat3 protein with an increase of its phosphorylation at 48 and 72 h of Pi treatment [19]. Moreover, in our experimental conditions, 0.1 μM doxorubicin does not cause any obvious effect on Stat3 and Erk1/2 phosphorylation and protein levels in MDA-MB-231 cells for up to 12 h of treatment (data not shown).

Figure 5A shows that doxorubicin induced a time-dependent increase in Erk activity starting at 12 h of doxorubicin incubation (and declining after 48 h). Interestingly, the Doxo-induced Erk 1/2 activation was impaired in response to Pi. None of the treatments significantly affected the total amount of proteins examined.

As far as Stat3 is concerned, Figure 5A shows also that, according to previous evidence [27], doxorubicin treatment resulted in an increase of Stat3 phosphorylation starting at 24 h and more evident at 48 h. Notably, the co-treatment with Pi, as expected [19], caused a decrease of the total amount of Stat3 protein at 48 h and prevented, at least in part, the doxorubicin-induced Stat3 phosphorylation.

In Figure 5B, it is shown that, in contrast to MDA-MB-231 cells, no obvious changes on Stat3 and Erk 1/2 phosphorylation and protein levels can be seen in MCF-7 cells in response to Pi.

Overall, the above findings indicate that Pi treatment of MDA-MB-231 cells interferes with the modulation of Erk1/2 and Stat3 activation in response to doxorubicin and suggest that the Pi-mediated inhibition of Erk and Stat3 signaling is involved in the enhancement by Pi of doxorubicin-induced cytotoxicity in MDA-MB-231 cells.

### 2.6. Discussion

To date, there is no effective targeted-therapy for triple-negative breast cancer (TNBC), novel neoadjuvant treatment options for TNBC are limited and little substantial progress has been made in the past decade [3,4]. Cytotoxic chemotherapy represents the landmark of current treatment strategies for early and metastatic TNBC. In this regard, third generation chemotherapeutic agents, including anthracyclines and taxanes, are considered the most effective available tools [8].

Doxorubicin is an anthracycline antibiotic routinely combined with taxol-based chemotherapeutics, but also used as a single agent for the treatment of TNBC [8,26]. Doxorubicin is a DNA-damaging agent, and its cytotoxic action has been mainly related to inhibition of topoisomerase II and also to the production of reactive oxygen species [9]. Although doxorubicin exerts robust antitumor activity, the appearance of important dose-dependent side effects, such as cardiotoxicity, and the acquisition of drug-resistance severely limited its clinical effectiveness [9,11].

Therefore, novel combination chemotherapeutic strategies, in which one novel compound is added as a “sensitizer” to increase the therapeutic index of doxorubicin, would definitely benefit cancer patients and are strongly warranted.

Dietary supplements, phytotherapeutic agents and naturally-occurring molecules with antitumor activity and with the least toxicity to normal tissues are suggested as interesting candidates to be investigated for their possible synergistic efficacy in combination with antineoplastic drugs [12,15,38,39]. Inorganic phosphate (Pi) is a common dietary component and an essential nutrient to the living organism [16,40]. Interestingly, Pi is emerging as an important signaling molecule capable of modulating multiple cellular functions by altering signal transduction pathways, gene expression and protein abundance in many cell types [18,41].

To note, we recently demonstrated that Pi enhances the doxorubicin-induced cytotoxicity in osteosarcoma cells in a p53-dependent manner and a mechanism involving Erk1/2 downregulation [22,42].

So far, poor research has been directed at determining the consequences of elevated Pi on the behavior of breast cancer cells [43]. Previously, we described initial evidence of Pi acting as a novel signaling molecule capable of eliciting a strong antiproliferative action in triple-negative MDA-MB-231 breast cancer cells [19].

As part of our continuing effort to extend the knowledge on the role of inorganic phosphate as a “naturally-occurring molecule” acting also as a “sensitizer” to increase the therapeutic index of clinical antitumor drugs, in this study, we report that in the MDA-MB-231 human breast cancer cell line, a well-established model system of highly aggressive triple-negative breast cancer, Pi strongly induces sensitization to doxorubicin by apoptosis induction. We provide evidence that the enhancement of doxorubicin-induced cytotoxicity by Pi occurs via a mechanism involving Erk1/2 and Stat3 downregulation.

Stat3 belongs to a family of latent cytoplasmic transcription factors that are activated by phosphorylation of a critical tyrosine residue, dimerization and nuclear translocation, upon binding of cytokines and growth factors to their membrane receptors [44].

Multiple lines of evidence place Stat3 at a central node in the development, progression and maintenance of many human tumors based on its ability to transactivate a number of genes whose products are involved in cell proliferation and survival, invasion, metastasis and angiogenesis. Constitutive activation of Stat3 is a frequent occurrence in breast tumors and has been demonstrated in a number of breast cancer cell lines [35]. Thus, Stat3 has been validated as an anticancer target in several contexts [45,46]. In addition, high levels of Stat3 activity have also been found to predict intrinsic drug resistance to chemotherapy [47], and Stat3 inhibition has been evaluated as a possible strategy to increase tumor cell response to cytotoxic agents [27,48,49,50,51].

Interestingly, in our experiments, we have found that, according to previous evidence [27], doxorubicin treatment results in an increase of Stat3 phosphorylation starting at 24 h and more evident at 48 h in MDA-MB-231 triple-negative breast cancer cells. Notably, the co-treatment with Pi caused a decrease of the total amount of Stat3 protein at 48 h and prevented, at least in part, the doxorubicin-induced Stat3 phosphorylation.

The Ras/Raf/Erk signaling pathway has been extensively studied over the past few decades. During this time, there have been significant breakthroughs in the discovery of interacting pathway components and insights into how mutations of these components can lead to aberrant signaling, uncontrolled proliferation and, in some cases, resistance to chemotherapy [36]. Research has also led to the development of inhibitors that specifically target critical elements of this pathway [52].

Notably, the Ras/Raf/Erk signaling pathway is relevant to breast cancer and is frequently activated, and a blockade of such a signaling pathway is considered a relevant strategy for therapeutic intervention [52,53,54]. Moreover, in several types of cancers, including breast cancer, Erk1/2 are upregulated in response to DNA-damaging chemotherapeutic agents, such as doxorubicin [28,29,36,37]. Activation of the ERK pathway by DNA-damaging agents has been described to correlate with increased apoptosis or to lead to an antiapoptotic effect.

Erk1/2’s role in promoting survival and progression into the cell cycle in the absence of DNA damage is well established [55]. Furthermore, several studies have implicated ERK activity in these same pro-survival and cell cycle progression responses in cells under DNA damaging conditions. In contrast, a small, but growing number of studies has indicated roles for Erk in the promotion of cell death under a variety of conditions, including following DNA damage [36,56].

Here, we have found that in triple-negative MDA-MB-231 breast cancer cells, Pi inhibits the Erk1/2 activation in response to low doses of doxorubicin; these data are consistent with a pro-survival, anti-apoptotic effect of doxorubicin-induced Erk1/2 activation in MDA-MB-231 cells.

Interestingly, our findings are fully in agreement with those indicating that Ras/Raf/Erk activation is related to resistance to doxorubicin in breast cancer cells and that interactions between doxorubicin and targeted therapy to inhibit the Ras/Raf/Erk pathway can enhance the induction of apoptosis and increase the effectiveness of chemotherapeutic drugs to induce cell death [57,58,59,60,61].

Our data clearly indicate that Pi can greatly enhance doxorubicin-induced cell death by affecting Erk1/2 and Stat3 signaling pathways within the tumor cells themselves.

Interestingly, in contrast to MDA-MB-231 cells, the enhancement of the cytotoxic activity of doxorubicin by Pi does not occur in MCF-7, T-47D and ZR-75-1 breast cancer cells (that are not “triple-negative” and express estrogen and progesterone receptors), strongly suggesting that Pi can have discrete effects depending on the “triple-negative receptor” status.

In this context, a very interesting hypothesis is that the difference of the MDA-MB-231 and MCF7 proliferation response to PI/Doxorubicin treatment could be, at least partially, due to the relation between the estrogen receptors’ status and cell cycle protein expression. In this regard, it is well known that cell cycle dysregulation plays an important role in breast cancer, and there is clear evidence that the estrogen receptor pathway plays a critical role in cell cycle dysregulation in breast cancer [62,63].

However, we do know that our data have to be considered “hypothesis generating”, rather than definitive. In this regard, the detailed molecular mechanism underlying this enhancement of doxorubicin-induced cytotoxicity by Pi remains unclear and is actively under our investigation.

Numerous recent data have strengthened a long-established hypothesis that a phosphate-sensing mechanism would detect changes in serum or local phosphate concentration and would inform the body, the local environment or the individual cell [41].

Importantly, so far, we are accumulating evidence that Erk1/2 and Stat3 inhibition in response to Pi is accompanied by changes of relevant protein levels, including B-Raf and Raf-1 kinases, and of intracellular ATP content (data not shown), and these events are actually under our investigation to further explore and explain how Pi acts as a signaling molecule and enhances doxorubicin-induced cytotoxicity.

Note that whatever the exact mechanism(s), here, we report that Pi may act as a potent enhancer of doxorubicin-induced cytotoxicity in triple-negative breast cancer cells.

Combination chemotherapy has received more attention in order to find compounds that could increase the therapeutic index of clinical anticancer drugs. Pi is emerging as an attractive candidate to be further investigated.

Importantly, in our study, Pi was found to have a positive pharmacological interaction, even with a very low dose (0.1 μM) of doxorubicin, that is expected to be more tolerable and associated with minimal undesired side-effects in patients, thus increasing the potential clinical relevance of our data.

New drug delivery systems containing calcium phosphate nanoparticles have been developed. Very interestingly, the release of inorganic phosphate from hydroxyapatite nanoparticles and its retention at local sites are known to occur, thus affecting Pi concentrations locally [64,65].

In addition, keeping in mind that phosphate is the most abundant anion in the cell with a high intracellular concentration (of about 100 mmol/L), it is easy to imagine that an increase of extracellular Pi can be found in the tumor microenvironment upon release from death cells during chemotherapy, contributing to amplifying the activity of chemotherapeutic agents.

## 3. Experimental Section

### 3.1. Materials

All cell culture materials were from Gibco-Life Technologies (Gaithersburg, MD, USA). Anti-tubulin antibodies were obtained from Oncogene-Calbiochem (La Jolla, CA, USA). Anti-*p*-ERK antibodies were obtained from Cell Signaling Technology (Danvers, MA, USA). All other antibodies were obtained from Santa Cruz Biotechnology (San Diego, CA, USA).

### 3.2. Cell Culture and Treatments

The human breast carcinoma cell lines MDA-MB-231, MCF-7, ZR-75-1 and T-47D were obtained from American Type Culture Collection (Rockville, MD, USA). MDA-MB-231 and MCF-7 cells were grown in Dulbecco’s Modified Eagle’s Medium (DMEM); T-47D and ZR-75-1 cells were grown in Roswell Park Memorial Institute (RPMI) medium. All media were supplemented medium with 2 mM glutamine, 100 U/mL penicillin, 100 mg/mL streptomycin and 10% fetal bovine serum (FBS) and cultured at 37 °C in a 5% CO_2_ humidified atmosphere. Unless noted, all experiments were done in the above medium, which contains 0.916 mM Pi, and concentrations listed in the figures are final Pi medium concentrations. Pi was added in the form of NaPO_4_, pH 7.4, from Sigma (Sigma-Aldrich Co, St. Louis, MO, USA) [23]. Doxorubicin was dissolved in ddH_2_O, stored at 4 °C and diluted with culture medium to final concentrations indicated in the figures [22]. Typically, subconfluent cells were split (5 × 10^5^/10 cm plate) and grown in 10% serum containing medium. After 24 h, the medium was removed, the cells were washed with PBS and incubated with 10% FBS fresh medium (Time 0), supplemented or not with Pi and doxorubicin, alone or in combination, and grown for the times and at concentrations indicated in the figures. Floating cells were recovered from culture medium by centrifugation, and adherent cells were harvested by trypsinization. Both floating and adherent cells were used in experiments aimed to study the expression of proteins involved in apoptosis and to perform FACS analysis [31].

### 3.3. Cell Viability Assay

Viable cells were determined by the 3-[4,5-dimethylthiazol-2-yl]-2,5-diphenyltetrazolium bromide (MTT) assay, as previously described [31]. Briefly, cells were seeded in 96-multiwell plates at a density of 4 × 10^3^ cells/well and grown in 10% serum containing medium. After 24 h, the medium was removed, cells were washed with PBS and cultured again in 10% serum fresh medium supplemented or not (control) with Pi (time 0) for up to 72 h (see the figure legends). Before harvesting, 100 μL of MTT solution (5 mg/mL) were added to each well and incubated at 37 °C for 3 h; then, the formazan product was solubilized by the addition of 100 μL 0.04 N HCl isopropanol. The optical density of each sample was determined by measuring the absorbance at 570 nm *vs.* 650 nm using an enzyme-linked immunosorbent assay reader (Molecular Device). Cell proliferation assays were performed three times (in replicates of 6 wells for each data point in each experiment). Data are presented as the means ± standard deviation for a representative experiment.

### 3.4. Evaluation of Cell Cycle Phases by Flow Cytometry

After Pi treatment, cells were recovered as described in Section 3.2, fixed by resuspension in 70% ice-cold methanol/PBS and incubated overnight at 4 °C. After fixing, samples were pelleted at 400× *g* for 5 min, and pellets were washed once with ice-cold PBS and centrifuged for a further 5 min. Pellets were resuspended in 0.5 mL DNA staining solution (50 μg/mL of propidium iodide, PI, and 100 μg RNase A in PBS) and incubated at 37 °C for 1 h in the dark. Samples were transferred to 5-mL Falcon tubes and stored on ice until assayed. Flow cytometric analysis was performed using a FACSCalibur flow cytometer (Becton Dickinson, San Jose, CA, USA) interfaced with a Hewlett-Packard computer (Model 310) for data analysis performed with the ModiFIT Cell Cycle Analysis software. For the evaluation of intracellular DNA content, at least 20,000 events for each point were analyzed, and regions were set up to acquire quantitative data of cells that fell into the normal G1, S, G2 regions and with fragmented DNA (sub-G1 or apoptotic events) [22,31].

### 3.5. Preparation of Cell Lysates

Cell extracts were prepared as follows. Briefly, 3 to 5 volumes of RIPA buffer (PBS, 1% NP-40, 0.5% sodium deoxycholate, 0.1% SDS) containing 10 μg/mL aprotinin, leupeptin and 1 mM phenylmethylsulfonyl fluoride (PMSF) were added to recovered cells. After incubation on ice for 1 h, samples were centrifuged at 18,000× *g* in an Eppendorf microcentrifuge for 15 min at 4 °C, and the supernatant (SDS total extract) was recovered. Some aliquots were taken for protein quantification according to the Bradford method [66]; others were diluted in 4× Laemmli buffer, boiled and stored as samples for immunoblotting analysis.

### 3.6. Immunodetection of Proteins

Typically, we employed 20 to 40 μg of total extracts for immunoblotting. Proteins from cell preparations were separated by SDS-PAGE and transferred onto nitrocellulose sheets (Schleicher & Schuell, Dassel, Germany) by a Mini Trans-Blot apparatus from Bio-Rad (Hercules, CA, USA). II goat anti-rabbit or anti-mouse antibodies, conjugated with horseradish peroxidase (BioRad), were used as a detection system the enhanced chemiluminescence (ECL), according to the manufacturer’s instructions (Amersham Biosciences, Buckinghamshire, UK).

### 3.7. Statistical Analysis

Experiments were performed three times with replicate samples, except where otherwise indicated. Data are plotted as the mean ± SD (standard deviation). The means were compared using analysis of variance (ANOVA) plus Bonferroni’s *t*-test. *p*-values of less than 0.05 were considered significant. National Institutes of Health ImageJ 1.42Q (NIH, Bethesda, MD, USA) software was used for densitometric analysis.

## 4. Conclusions

Our findings that inorganic phosphate, a very simple “naturally-occurring molecule”, can have antiproliferative effects on TNBCs and achieve additive cytotoxic effects when combined with doxorubicin illustrates its potential for clinical applications. It must be pointed out that by our data, it is absolutely not suggested that the Pi concentration should be increased at the systemic level to treat breast cancer (since it causes unfavorable effects in physiological functions), but just that upregulating Pi levels in the extracellular environment at local sites (such as breast and bone, for example) for brief periods might contribute to the development of novel and inexpensive modalities for therapeutic intervention in some tumors, including triple-negative breast cancer.
